# Unpacking the Mediating Role of Burnout on the Relationship Between Resilience and Engagement in Chinese Senior High School Students’ English Learning

**DOI:** 10.3390/bs16101714

**Published:** 2026-09-22

**Authors:** Meina Li, Dan Lu, Honggang Liu

**Affiliations:** 1 School of International Studies, Northeast Normal University, Changchun 130024, China; 2 School of International Studies, Soochow University, Suzhou 215006, China

**Keywords:** English learning resilience, English learning burnout, English learning engagement, mediation model

## Abstract

Resilience, burnout and engagement are critical emotional factors influencing language learning, yet existing studies often overlook their combined effects and the interaction between them. This study aimed to explore the relationships among resilience, burnout, and engagement in English learning among senior high school students. A sample of 637 senior high school students from southeastern China completed validated questionnaires, and ten participants were subsequently recruited for semi-structured interviews. Quantitative data were analyzed using SPSS 26 and Mplus 8.3, while interview transcripts were subjected to thematic analysis. The results indicated that participants demonstrated relatively high levels of resilience and engagement alongside low levels of burnout. Structural equation modeling revealed that resilience positively predicted engagement and negatively predicted burnout. Critically, of the two dimensions of burnout, only demotivation exerted a significant negative predictive effect on engagement and served as a partial mediator between resilience and engagement, whereas exhaustion played no significant mediating role. Qualitative findings corroborated these quantitative patterns, illustrating that resilient learners sustained engagement by counteracting motivational decline. These findings yield pedagogical implications for emotion regulation in examination-oriented EFL contexts.

## 1. Introduction

Driven by the expansion of positive psychology in foreign language education, which emphasizes the significance of positive learner factors (Dewaele et al., 2019; MacIntyre & Gregersen, 2012), research in foreign language education has shifted its emphasis from learners’ negative personality traits and emotions toward positive ones (Wang et al., 2021; H. Liu et al., 2026b). Following this paradigm shift, scholarly attention has increasingly centered on exploring how learners mobilize positive psychological resources to cope with academic stress and sustain adaptive learning engagement. Adopting this theoretical perspective, this study focuses on Chinese senior high school students to explore their positive psychological mechanisms under academic pressure.

Given the inherent challenges of language learning, English-as-a-foreign-language (EFL) learners are at risk of experiencing burnout related to English learning (H. Liu et al., 2024). For Chinese EFL learners, the demands are particularly heightened, as English is a core subject in the National College Entrance Examination (Gaokao), a high-stakes test that shapes students’ university admission and long-term prospects (Fan et al., 2024; Wu et al., 2024). In this exam-oriented context, where English learning is driven primarily by external test requirements, students are subjected to sustained intensive academic pressure. This chronic external pressure can gradually deplete students’ emotional resources and undermine their motivation for English learning, a motivational decline that constitutes a core symptom of burnout and precipitates reduced learning engagement. Under such pressure, resilience and burnout stand out as particularly influential. Resilience empowers students to recover from setbacks in English learning and sustain active engagement; burnout, by contrast, manifests as emotional exhaustion and diminished willingness to invest effort in English learning.

Existing research has established that resilience negatively predicts burnout and positively predicts engagement, whereas burnout negatively predicts engagement. However, the mechanism through which resilience relates to engagement via burnout remains underexplored, particularly among Chinese senior high school students facing intensive English learning demands. Although recent studies have examined these relationships in Chinese EFL contexts (e.g., Wu et al., 2024; H. Liu et al., 2023), the mediating process has yet to be fully articulated within Control-Value Theory (CVT; Pekrun, 2006). Drawing on CVT, the present study proposes a mediation model to elucidate how positive psychological resources operate under sustained academic stress. In light of the Gaokao-driven pressures outlined above, elucidating this mechanism would advance theoretical understanding of the resilience-engagement pathway and offer practical guidance for mitigating burnout and sustaining engagement in this population.

## 2. Literature Review

### 2.1. Theoretical Foundation for the Study

The present study adopts CVT as the theoretical framework for analyzing the mechanisms underlying resilience, burnout, and engagement. CVT offers an integrative framework for understanding achievement emotions in educational settings. The theory posits that antecedents, emotions, and academic outcomes are dynamically interconnected, forming a reciprocal causal system that has informed much of the subsequent research on academic emotions.

The core premise of CVT is that achievement emotions are primarily elicited by two types of cognitive appraisal: control appraisals and value appraisals. Control appraisals encompass learners’ subjective sense of agency over academic tasks and outcomes, for instance, whether they feel capable of mastering the material. Value appraisals capture the perceived significance or practical worth of learning activities, such as how relevant or beneficial a task appears. These appraisals, in turn, are shaped by more distal social antecedents and individual antecedents, such as personality traits and contextual factors (Pekrun & Perry, 2014; Harley et al., 2019). It should be noted that while control and value appraisals are central to CVT, they are employed in the present study as a conceptual lens for interpreting the hypothesized mediation mechanism, rather than as directly measured variables in the empirical model. Through these appraisal mechanisms, distal antecedents indirectly influence the arousal of achievement emotions (Pekrun et al., 2007), which in turn shape academic outcomes. This antecedent-appraisal-emotion-outcome chain provides a robust framework for the present study. Specifically, resilience can be conceptualized as a distal individual antecedent, burnout as a negative achievement emotion, and engagement as a key academic outcome within this CVT framework. The specific relationships among these variables will be elaborated in the following sections.

### 2.2. Resilience and Engagement in English Learning

In the context of foreign language education, resilience is generally understood as a personal attribute that equips learners to persist through difficulties (Shin & Kim, 2017; Kim & Kim, 2017; H. Liu & Han, 2022; Chu et al., 2024). Kim and Kim (2017), for instance, conceptualized L2 resilience as an inherent quality that enables learners to not only rebound from academic setbacks but also maintain their momentum even in the face of persistent adversity. Expanding on this view, Shin and Kim (2017) further emphasized the role of inner strength, describing resilience as a psychological resource that helps learners overcome the specific challenges associated with learning English. This capacity is consequential for learner behavior and outcomes: learners with high resilience are less likely to withdraw from demanding language tasks and tend to sustain higher levels of learning participation and engagement (W. Liu et al., 2022; Zarrinabadi et al., 2022) and maintain academic achievement (H. Liu et al., 2026a).

In foreign language education, the relationship between resilience and engagement has attracted growing attention, although most studies have centered on university students. For instance, Zarrinabadi et al. (2022) identified resilience as a critical personal predictor of engagement and well-being among 300 Iranian university EFL learners. Within the Chinese context, W. Liu et al. (2022) assessed the influence of resilience and mindfulness on 1693 Chinese EFL learners’ learning engagement, finding that both resilience and mindfulness served as meaningful predictors of engagement. Similarly, Wang et al. (2025) found that L2 resilience was positively associated with all four dimensions of engagement among 649 Chinese EFL undergraduates.

Despite these contributions, empirical research addressing the interplay between resilience and engagement among Chinese senior high school students remains scarce. The present study aims to address this gap by investigating how resilience influences engagement in Chinese senior high school English learning from the perspective of CVT. According to CVT, the relationship between resilience and engagement in English learning is not a simple direct association but involves a layered, progressive mechanism of internal processes. Accordingly, English learning resilience, as a stable personality trait, can be conceptualized as a key distal individual antecedent that may influence both control and value appraisals, thereby potentially affecting engagement through its impact on emotions.

Before examining these indirect pathways, we first test the direct effect of resilience on engagement as the necessary baseline condition for the mediated model. Therefore, building upon the aforementioned empirical evidence and theoretical support, we hypothesize:

**H_1_.**  *English learning resilience can positively and directly predict English learning engagement, encompassing both its behavioral and agentic dimensions.*

### 2.3. Burnout and Resilience in English Learning

Among the wide range of emotional experiences accompanying English learning, negative emotions can be particularly detrimental (H. Liu et al., 2026b). Students with low intrinsic motivation for English may perceive it as a burden, gradually experiencing what H. Liu and Zhong (2022) describe as “a negative, complex psychological state where learners feel demotivated by their confidence and interest and even exhausted regarding English learning.” This emotional state is known as English learning burnout (ELB). Within this operationalization, ELB is treated as an affective construct rather than a purely motivational syndrome. As a manifestation of negative academic emotions, ELB functions as a psychological stressor that significantly and negatively predicts engagement (H. Liu et al., 2023, 2025a) and impairs their academic achievement (Pham Thi & Duong, 2024). However, learners vary in their susceptibility to such emotional exhaustion, and resilience may account for this variation.

According to CVT, learners’ personal traits influence their academic emotions through control and value appraisals. Specifically, students with higher English learning resilience are better able to maintain a sense of control and value recognition when facing difficulties, thereby reducing negative emotions, such as burnout. In contrast, learners with lower resilience are more likely to lose control and devalue learning after setbacks, which can trigger or worsen burnout. This theoretical reasoning is consistent with existing empirical evidence. H. Liu et al. (2024) recruited 334 senior high school students to explore the complex interplay among students’ burnout, resilience and academic achievement in English learning. The findings showed a significant negative correlation between high school students’ English learning burnout and resilience. Similarly, Wu et al. (2024), in a mixed-methods study of 413 Chinese senior high school EFL learners, found that students’ English learning burnout was negatively associated with their English learning resilience. While these findings confirm the directional relationship, the conceptualization of resilience as an antecedent to burnout is grounded in CVT. Therefore, building upon the aforementioned empirical evidence and theoretical support, we hypothesize:

**H_2_.**  *English learning resilience can negatively and directly predict English learning burnout, encompassing both its exhaustion and demotivation dimensions.*

### 2.4. Engagement and Burnout in English Learning

English learning demands considerable time and persistent effort (H. Liu et al., 2025b). Such proactive student participation in learning activities is conceptualized as academic engagement (Fredricks et al., 2004; Reeve, 2012). Originating from general education research, engagement is widely viewed as a multifaceted construct in foreign language education, yet scholars have not agreed on its specific dimensional divisions (Christenson et al., 2012). A widely accepted model by Fredricks et al. (2004) proposes three dimensions: behavioral, emotional, and cognitive engagement. Reeve and Tseng (2011) later added a fourth dimension, agentic engagement, based on Self-Determination Theory. However, empirical studies have noted considerable conceptual overlap among these dimensions (Vo, 2024). Given that different dimensions often intertwine in practice, a two-dimensional structure, comprising behavioral and agentic engagement, has been proposed to capture the core features of engagement more parsimoniously (e.g., H. Liu et al., 2025b; Cai & Liu, 2025).

Although engagement is widely regarded as a strong predictor of long-term academic success (e.g., Skinner et al., 1998; Y. Guo et al., 2023), it can be undermined by negative emotions like ELB (Pekrun et al., 2002). In foreign language education, Wu et al. (2024) confirmed that high burnout often coincides with low engagement, and H. Liu et al. (2025a) demonstrated that English learning burnout negatively predicts engagement. Collectively, these findings indicate that while engagement positively predicts academic achievement, burnout may inhibit engagement. This negative relationship can also be clearly explained within the framework of CVT. According to CVT, negative academic emotions may arise when learners perceive an imbalance between their sense of control and the value they attach to learning activities, and these emotions in turn shape learning behaviors. English learning burnout, as a typical negative emotion characterized by exhaustion and demotivation in learning, significantly undermines learners’ initiative and persistence, thereby reducing engagement. Conversely, when the level of burnout is low, learners are able to maintain a more positive and stable emotional state, which in turn supports higher levels of learning engagement. Therefore, from the CVT perspective, English learning burnout exerts a direct negative predictive effect on English learning engagement. Therefore, we hypothesize:

**H_3_.**  *English learning burnout can negatively and directly predict English learning engagement, at both the construct level and across the dimensions of burnout (exhaustion and demotivation) and engagement (behavioral and agentic engagement).*

### 2.5. The Mediating Role of Burnout Between Resilience and Engagement

Drawing upon the appraisal-emotion-achievement framework of CVT along with the conceptualization of resilience, burnout and engagement, a preliminary attempt to hypothesize the links between the three psychological variables among EFL learners can be made. Resilience can be regarded as a critical personality trait in language learning (H. Liu & Han, 2022), categorized as a distal individual antecedent within the CVT framework. According to this theory, individual antecedents exert their influence by shaping students’ control-value appraisals. Specifically, high-resilience individuals tend to appraise complex English learning tasks as surmountable challenges rather than uncontrollable threats. This positive cognitive reappraisal maintains their higher sense of academic control. Conversely, students with low levels of resilience often succumb to learned helplessness, forming a negative appraisal pattern characterized by a low sense of control and value. This negative cognitive evaluation could theoretically induce burnout, a negative achievement emotion manifested as exhaustion, demotivation and a diminished sense of accomplishment (H. Liu & Zhong, 2022). Furthermore, to prevent further depletion of psychological energy, students in a state of burnout instinctively reduce their cognitive and emotional investment in learning activities, leading to a significant decline in learning engagement, which has been regarded as a key educational outcome (Reeve & Tseng, 2011; Wang et al., 2024; Zhao et al., 2025). Consequently, the logical chain derived from CVT suggests that resilience (antecedent) mitigates burnout (emotion) through control and value appraisals, which in turn preserves or enhances engagement (outcome) in English learning. Therefore, we hypothesize:

**H_4_.**  *Burnout mediates the relationship between resilience and engagement in English learning, at both the construct level and across the dimensions of burnout (exhaustion and demotivation) and engagement (behavioral and agentic engagement).*

### 2.6. The Conceptual Model

Although existing literature has confirmed the associations between these variables to some extent, existing research scarcely delves into the specific educational context of Chinese EFL to systematically analyze how these three variables interact under the unique influence of Confucian cultural traditions and a rigorous examination system. Moreover, existing studies on English learning resilience disproportionately focus on university students (e.g., Ghanizadeh et al., 2019), while attention to high school students is scarce. This gap is noteworthy because this group operates under the intense pressure of Gaokao, characterized by high-stakes requirements and heavy academic burdens (Fan et al., 2024; Wu et al., 2024). Such a stressful learning environment may intensify burnout and undermine learners’ engagement. Thus, exploring how resilience helps high school students withstand burnout and maintain engagement under exam pressure holds urgent practical significance.

Drawing on the aforementioned theoretical and empirical foundations and the identified research gap, the present study proposes a hypothetical model (see Figure 1) wherein ELB mediates the relationship between ELR and ELE. Specifically, ELB comprises two dimensions: demotivation and exhaustion, while ELE consists of behavioral engagement and agentic engagement. The model hypothesizes that ELR not only directly predicts the two dimensions of ELE but also exerts an indirect effect on them through the two dimensions of ELB.

## 3. Methodology

### 3.1. Participants

For the quantitative phase, 637 valid questionnaires were collected from EFL students across five public senior high schools in southeastern China, covering all grades from Senior One to Senior Three. Of the 700 surveys distributed, incomplete, repetitive, and outlier responses were excluded, yielding a 91% valid response rate. The sample included 272 males (42.7%) and 365 females (57.3%), consisting of Grade 1 (39.3%), Grade 2 (43.0%), and Grade 3 (17.7%). The small share of Grade 3 participants was attributable to their heavy Gaokao revision schedules, which limited available time for questionnaire completion. All students took compulsory English courses. Due to restricted access to schools, convenience sampling was adopted.

For interviews, ten students participated in semi-structured interviews lasting approximately 20 min each. Their demographic backgrounds and subscale scores are presented in Supplemental Materials.

### 3.2. Instruments

The present study employed a composite questionnaire assembled from three existing validated scales, supplemented by a semi-structured interview protocol. All questionnaire items were scored on a 5-point Likert scale ranging from 1 (strongly not true for me) to 5 (strongly true for me). Internal consistency was assessed using Cronbach’s α, with values ≥ 0.70 considered acceptable (Hair et al., 2019). Construct validity was examined through confirmatory factor analysis (CFA) with maximum likelihood estimation, using the following fit criteria: CFI ≥ 0.90, TLI ≥ 0.90, RMSEA ≤ 0.08, and SRMR ≤ 0.10 (Hair et al., 2019). While the burnout and engagement scales were developed for English learning contexts, the resilience scale was adapted from Martin and Marsh (2006) to ensure conceptual consistency. All items were translated into Chinese and back-translated into English by two bilingual researchers, with discrepancies resolved through discussion.

#### 3.2.1. Questionnaire

Students’ English learning resilience was measured by six items (Q01–Q06) adapted from Martin and Marsh (2006). This unidimensional scale was selected for its concise format, consistent with the present study’s conceptualization of resilience. CFA confirmed the one-factor structure. The scale demonstrated good reliability (α = 0.908) and satisfactory model fit (CFI = 0.974, TLI = 0.957, RMSEA = 0.063, SRMR = 0.027). Additionally, students’ English learning burnout was assessed using the Senior High School English Learning Burnout Scale (SHSELBS; H. Liu & Zhong, 2022). The scale comprises 10 items categorized into two dimensions: exhaustion (4 items) and demotivation (6 items). CFA confirmed this two-factor structure. The scale demonstrated good reliability (α = 0.927) and satisfactory model fit (CFI = 0.942, TLI = 0.921, RMSEA = 0.078, SRMR = 0.047). Reliability for the two subscales was also satisfactory (exhaustion: α = 0.91, CR = 0.91; demotivation: α = 0.88, CR = 0.88). In addition, students’ English learning engagement was examined through the ten-item English Learning Engagement Scale (Cai & Liu, 2025). The scale consists of 10 items equally divided into behavioral and agentic engagement subscales. Following CFA, Q24 (I will inform my English teacher about what interests me in learning English) was removed due to a high modification index suggesting cross-loading with other items in the engagement measure. Theoretically, this item may not fully capture agentic engagement in the Chinese senior high school EFL context, where classroom norms may not always encourage students to proactively communicate their learning interests to the teacher. This yielded a final two-factor structure: behavioral engagement (Q17–Q21) and agentic engagement (Q22–Q23, Q25–Q26). The refined scale demonstrated good reliability (α = 0.926) and satisfactory model fit (CFI = 0.965, TLI = 0.950, RMSEA = 0.072, SRMR = 0.042). Subscale reliabilities were adequate (behavioral engagement: α = 0.92, CR = 0.92; agentic engagement: α = 0.89, CR = 0.88). The detailed questionnaire items are shown in Appendix A. 

#### 3.2.2. Interview Protocol

To complement and enrich the quantitative findings, semi-structured interviews were conducted. The interview protocol was informed by both the existing literature and the preliminary quantitative results, with a particular focus on exploring how students experience the interplay among resilience, burnout, and engagement. Open-ended questions were used, and all interviews were carried out in Chinese to facilitate full and accurate expression. The sequence and number of questions were adapted flexibly based on each participant’s responses. The interview protocol, including the main guiding questions, is provided in Appendix B.

### 3.3. Data Collection

This study followed an explanatory sequential mixed-methods design (Creswell & Plano Clark, 2018). Quantitative data were collected and analyzed prior to the qualitative interviews, which were conducted to contextualize the quantitative findings. A two-phase data collection procedure was followed. In the initial phase, a questionnaire was made available online through Wenjuanxing (https://www.wjx.cn) between September and October 2025. Ethical approval was obtained from the participating schools’ administration, and all students gave voluntary consent online before completing the survey. Before responding, they were informed that the survey was anonymous and that their data would be used solely for research. Following that, genuine gratitude was extended to the participants.

In the second stage, interviews involving ten students were conducted either online or offline, with each one averaging 20 min in length. Prior to the interview, individual interviews were conducted with each participant and audio-recorded with their consent. All sessions were carried out in Chinese, the students’ native language. The interview protocol was adapted flexibly according to the students’ responses and the surrounding context. Additionally, the interviewer noted and elaborated on key points to gain more profound insights into the students’ resilience, burnout, and engagement in English learning. After the interviews, the audio recordings were transcribed to assist the subsequent analysis. The transcripts were then organized and scrutinized. Ultimately, the findings from the qualitative analysis were employed to support further quantitative research.

### 3.4. Data Analysis

For the quantitative data analysis, no missing data were present, as only fully completed questionnaires were retained for analysis. Harman’s single-factor test indicated that common method bias was not a serious concern, as the first factor explained 44.06% of the total variance, below the recommended 50% threshold (Podsakoff et al., 2003). Univariate normality was confirmed with skewness and kurtosis within the recommended thresholds of |3.0| and |10.0|, respectively (Kline, 2016). Item analysis indicated satisfactory internal consistency and item discrimination for each subscale. Given that each scale was theoretically grounded, confirmatory factor analysis (CFA) was performed to validate the measurement models. In the main analysis, descriptive statistics and correlation analyses were first conducted to provide an overview of the data. Subsequently, the hypothesized structural equation model (SEM) was tested using 2000 bootstrap resamples with bias-corrected 95% confidence intervals to test mediating effects, which were deemed significant if the CI did not contain zero (Field, 2017). Model fit was evaluated using CFI, TLI, RMSEA, and SRMR, with acceptable thresholds set at CFI ≥ 0.90, TLI ≥ 0.90, RMSEA ≤ 0.08, and SRMR ≤ 0.10 (Hair et al., 2019).

For the qualitative data analysis, interview recordings were transcribed verbatim and analyzed using Kuckartz’s (2014) thematic analysis. Data were systematically coded using a deductive and inductive approach: initial coding around resilience, burnout, and engagement was guided by the research hypotheses and quantitative model, followed by secondary coding to extract subthemes. The recursive process involved moving between original text, codes, themes, and quantitative results. The interviews were conducted by the first author, who had no prior teaching or evaluative relationship with the participants. Chinese quotations were translated into English following translation and back-translation procedures. To ensure accuracy, transcripts and codes were returned to participants for confirmation. To ensure reliability, a second researcher independently coded 25% of randomly selected transcripts, with disagreements resolved through discussion among all three coders. Codes were iteratively reviewed and refined, and member checking was conducted by sharing preliminary thematic interpretations with several interviewees for feedback. The detailed coding results are provided in Supplemental Materials.

## 4. Results

### 4.1. Results of Primary Analyses

Descriptive statistics for resilience, burnout (exhaustion and demotivation), and engagement (behavioral and agentic engagement) are presented in Table 1.

Pearson correlation analysis revealed that resilience was significantly and positively correlated with both behavioral engagement (*r* = 0.53, *p* < 0.01) and agentic engagement (*r* = 0.51, *p* < 0.01). In contrast, the two sub-dimensions of burnout, exhaustion and demotivation, were significantly and negatively correlated with resilience (*r* = −0.49 and −0.45, respectively, *p* < 0.01) and with both behavioral engagement (*r* = −0.48 and −0.51, *p* < 0.01) and agentic engagement (*r* = −0.37 and −0.40, *p* < 0.01).

### 4.2. Results of SEM

To test our hypothesized model, we computed the SEM. Multicollinearity was assessed using VIF values; all were below 5, indicating no severe multicollinearity. The SEM (see Figure 2) generated a satisfactory model fit: CFI = 0.947, TLI = 0.939, RMSEA = 0.063, and SRMR = 0.048. The values met the cut-off scores (Hair et al., 2019).

As shown in Table 2 and Figure 2, the SEM analysis revealed several key findings on direct effects. First, ELR could significantly predict students’ ELE; thus, H_1_ was supported. Second, ELR could significantly predict students’ ELB; thus, H_2_ was supported. Third, among the dimensions of ELB, only demotivation was a negative and significant predictor; exhaustion showed no significant effect. These results provided partial support for H_3_. Above all, partial support was found for H_4_.

#### 4.2.1. Direct Effects

Regarding the direct effects, resilience is a negative and direct predictor of global burnout in English learning. More specifically, ELR negatively predicts exhaustion (β = −0.528, *p* < 0.001), and ELR negatively predicts demotivation (β = −0.505, *p* < 0.001). The results suggest that EFL students with higher levels of resilience are less susceptible to burnout.

The results of the complex SEM indicate that resilience could directly and positively predict engagement in English learning. Furthermore, from Table 2, it can be seen that resilience can predict behavioral engagement (β = 0.355, *p* < 0.001) and agentic engagement (β = 0.405, *p* < 0.001) directly.

Regarding the direct effects of the dimensions of English learning burnout, exhaustion did not exert a significant unique predictive effect on behavioral engagement directly (β = 0.066, *p* > 0.05) and agentic engagement (β = 0.123, *p* > 0.05) in this model; while demotivation could directly negatively predict behavioral engagement in English learning (β = −0.457, *p* < 0.01) and agentic engagement (β = −0.404, *p* < 0.01). Interviews with participants illustrated that burnout’s impact on engagement in English learning primarily centered on the relationship between demotivation and ELE.

#### 4.2.2. The Mediating Role of Burnout Between Resilience and Engagement

The indirect effect via demotivation was significant for behavioral engagement (β = 0.231, *p* < 0.01) and agentic engagement (β = 0.204, *p* < 0.01). The total effect of resilience on behavioral engagement was 0.551 (*p* < 0.001), with a direct effect of 0.355 (*p* < 0.001) and a total indirect effect of 0.196 (*p* < 0.001). For agentic engagement, the total effect was 0.544 (*p* < 0.001), with a direct effect of 0.405 (*p* < 0.001) and a total indirect effect of 0.139 (*p* < 0.001). The indirect effect via demotivation accounted for 41.9% and 37.5% of the total effects, respectively. The full model explained a substantial proportion of variance in behavioral engagement (R^2^ = 0.427) and agentic engagement (R^2^ = 0.371), indicating substantial effect sizes (Cohen, 1988). However, the indirect effect via exhaustion was not significant.

### 4.3. Results of Interview

To complement the quantitative findings, the interview data were analyzed to explore the processes underlying these relationships. The qualitative data illustrate how these variables operate at the experiential level.

As illustrated by Student 5 and Student 10, resilience appears to play a role in reducing burnout in English learning:

*In class, when I experience setbacks, my enthusiasm for English learning is indeed affected. On my last mock exam, I performed very poorly. During that period, I felt frustrated whenever I saw English papers and developed some resistance toward English learning, with decreasing motivation. However, I have reasonable confidence in my abilities and believe that I could master English through my own efforts. Therefore, I carefully organized my mistakes and conducted targeted practice based on my weak areas. My efforts eventually paid off. My English grades gradually improved and I became increasingly motivated and gained a sense of achievement.*

*(Student 5, high resilience, low burnout, 23 October 2025)*

*I used to think I could improve my English if I worked hard, but after failing several mock exams, I started to doubt myself. I don’t really believe I have the ability to get better anymore. Whenever I see a reading passage or a grammar task, I feel overwhelmed. I often feel tired and unmotivated, and I don’t see the point in trying. I just do the bare minimum to get by. I feel like I’m stuck and no matter what I do, it won’t make a difference.*

*(Student 10, low resilience, high burnout, 23 October 2025)*

Two contrasting cases illustrate how resilience differentiates learners’ experiences of burnout at the individual level. Student 5 experienced a setback but maintained confidence in her ability to improve, reflecting a high level of resilience. This resilience appeared to sustain her sense of control, as she explained, “I have reasonable confidence in my abilities and believed that I could master English through my own efforts.” This sense of control enabled her to take corrective action and restore motivation, thereby reducing her burnout. In contrast, Student 10 expressed doubt about his capacity to make progress, reflecting low resilience, and this low resilience was accompanied by a diminished sense of control and a perceived lack of value in his efforts, which contributed to feelings of being “unmotivated.” While the SEM results suggest an overall negative association between resilience and burnout, these interviews reveal the subjective processes that may underlie this pattern, namely, whether learners maintain a sense of control and value in the face of difficulty.

The qualitative data also substantiated the essential role of resilience in enhancing engagement in English learning:

*By not giving up and persisting with sustained effort, I successfully tackled those tricky grammar problems in English learning. This also made me like learning English more. Currently, in addition to taking detailed notes and actively responding to my teacher’s questions in class, I also proactively seek out English papers to work on after school.*

*(Student 1, high resilience, high engagement, 23 October 2025)*

Student 1’s interview illustrates the process through which resilience may translate into sustained engagement. When faced with learning challenges, he persisted with sustained effort rather than withdrawing. This persistence reflects a resilient mindset that enables learners to maintain engagement despite difficulties. This temporal sequence, from facing a challenge, to persisting, to regaining confidence, and ultimately to initiating extra practice, offers a complementary perspective on the resilience-engagement pathway.

Consistent with the quantitative findings, the interview data indicated a negative effect of demotivation on engagement, whereas exhaustion did not exert a significant influence. As indicated in the following interview excerpt:

*Although I do not feel particularly exhausted, I sometimes lack energy and motivation in my English learning. This has definitely affected my normal English study. For example, I used to get low scores in English listening. Even if I worked hard, I did not feel much progress. So I gradually lost my enthusiasm later, and even developed resistance to English listening. I did not want to do listening exercises, for fear that I would make many mistakes again.*

*(Student 3, low burnout, moderate engagement, 23 October 2025)*

As illustrated by Student 3, demotivation manifested primarily through diminished self-efficacy and a loss of interest. Student 3’s experience highlights how perceived incompetence (e.g., in listening tasks) breeds helplessness, directly curbing the willingness to participate. Crucially, this narrative clarifies why demotivation, rather than exhaustion, served as the primary constraint on engagement. Exhaustion typically implies a state of being “worn out” or having depleted energy resources. However, Student 3 did not describe being too tired to learn; rather, she described a deficit in value and expectancy. Thus, the reduced engagement was a result of demotivation rather than exhaustion.

A different pattern was observed in another case. Despite reporting a moderate level of burnout and feeling tired at times, this student maintained high engagement in English learning. As he explained:

*I do feel tired sometimes, especially with all the exams and homework. But since I was little, my parents and teachers always told me that if you want to get good results, you have to work hard. So even when I’m tired, I still do my English assignments and try to pay attention in class. I just think I have to keep going.*

*(Student 9, moderate burnout, high engagement, 23 October 2025)*

Although Student 9 acknowledged feeling tired, his sustained engagement appeared to be driven by a belief that effort and perseverance are necessary for academic success, a value he had internalized from an early age. This possibility is not fully illuminated in the quantitative model alone. This culturally shaped dynamic is made visible through the qualitative data, offering a complementary explanation for why exhaustion did not significantly predict engagement in the overall sample.

This mediating role of demotivation was also reflected in the qualitative data. As illustrated in the following excerpt, although the student did not explicitly articulate that demotivation played a mediating role, his narrative clearly suggested that resilience influenced engagement by alleviating demotivation:

*I have always believed that there are always more solutions than problems, I feel confident that I can find a way to solve any challenge I encounter. For example, last month, I struggled with English reading comprehension. Gradually, I began to lose motivation and felt that no matter how hard I tried, it was useless; I didn’t even want to touch the exercises anymore. However, I soon realized I couldn’t just give up. After adjusting my mindset, I regained motivation. I persisted in carefully summarizing my mistakes and analyzing where my understanding had gone wrong. Eventually, my accuracy improved significantly. This success regained my motivation to invest more time and energy into learning English.*

*(Student 2, high resilience, low burnout, high engagement, 23 October 2025)*

Student 2’s experience illustrates the mediating process through which resilience appears to sustain engagement. When his reading comprehension declined despite effort, he “gradually lost motivation” and felt his effort was “useless.” Crucially, his resilience was activated to counter this state. He adjusted his mindset and “regained motivation.” This account suggests that resilience may not directly produce engagement; rather, it appears to alleviate demotivation, which in turn is associated with renewed engagement.

## 5. Discussion

This study aimed to investigate the relationship between ELR and ELE, with ELB examined as the mediating mechanism. Of the four hypotheses proposed, two received partial support, while two received full support. The following section provides a detailed discussion of these results.

### 5.1. Relationships Among Resilience, Burnout and Engagement in Students’ English Learning

Firstly, the results suggest that EFL students with higher levels of resilience are less susceptible to burnout, echoing previous studies (Fernández-Castillo & Fernández-Prados, 2023; H. Liu et al., 2023, 2024; Wu et al., 2024). Among them, several studies conducted in the Chinese context have documented a negative association between resilience and burnout. H. Liu et al. (2024), for example, reported that students with higher levels of academic resilience tended to experience lower levels of academic burnout in English learning.

In addition, resilient students tend to be more engaged in their learning, which is supported by sporadic studies conducted in general education that found that students who had higher levels of confidence and coping skills in their academic tasks were more likely to be involved and interested in their learning activities (Romano et al., 2021; Versteeg et al., 2022). In foreign language education, a few scholars have also explored the relationship between resilience and engagement in English learning, revealing that resilience positively impacts engagement in English learning. For instance, H. Liu et al. (2023) identified a positive predictive role of resilience on engagement in English learning. Similarly, Wu et al. (2024) confirmed the positive association between resilience and engagement, reporting that high levels of resilience are often accompanied by more engagement.

Additionally, the results suggest that EFL students with lower levels of demotivation are more likely to engage behaviorally and agentically in English learning. Demotivation is often manifested as a loss of interest in English and a lack of confidence in completing English learning tasks (H. Liu & Zhong, 2022). When students experience demotivation, their interest in learning tasks and intrinsic motivation significantly decrease. This weakening of intrinsic motivation is associated with their behavioral and agentic engagement in learning activities. For example, a student who was initially interested in English learning may reduce involvement in class if they become demotivated due to long-term boredom with the learning content or a belief that they are unable to complete the learning tasks. Although some scholars have revealed the negative relationship between certain dimensions of engagement and burnout in the field of general education (Widlund et al., 2023), scant research has delved into the relationship between demotivation, as a subdimension of burnout, and engagement, especially in the domain of foreign language education. This study aims to address the research gap by investigating the impact of students’ burnout on engagement from the perspective of demotivation in their English learning.

However, the results of this study revealed that exhaustion did not demonstrate a significant unique predictive effect on engagement in this model, a finding that diverges from some prior studies reporting significant negative associations between exhaustion and engagement (e.g., Cai & Liu, 2025). While our data do not permit a definitive explanation for this discrepancy, several tentative interpretations may be considered in light of sample characteristics and contextual factors. First, the level of exhaustion observed in this study was relatively low. It is possible that for the majority of high school students in this sample, exhaustion had not reached a threshold sufficient to exert a meaningful impact on their engagement. In essence, they infrequently experienced physical or emotional exhaustion, which might reduce the likelihood of this state affecting their English learning engagement. Second, the non-significant association between exhaustion and engagement might partly stem from the voluntary nature of participation. It is conceivable that students experiencing high levels of exhaustion may have been less inclined to volunteer for the study on top of their already demanding academic workload, potentially yielding lower overall exhaustion levels within the sample and making a significant effect harder to detect. Third, contextual-cultural factors offer another tentative account. In the Chinese context, learners are inclined to view perseverance in the face of academic hardship as a moral and self-cultivation practice (Li, 2005). This culturally ingrained belief, historically reinforced by the imperial examination system, continues to shape students’ attitudes toward academic effort in the contemporary Gaokao context (Tian, 2018). Senior high school students may therefore exhibit a higher tolerance threshold for exhaustion, as persistence in the face of difficulty is understood as a normatively expected behavior rather than an exceptional effort. This interpretation is tentatively supported by our qualitative data, where Student 9, despite reporting moderate burnout, still maintained high engagement. This echoes recent evidence that culturally framed negative emotions can also assume motivational functions in Chinese EFL contexts (G. Guo et al., 2026).

### 5.2. The Mediating Role of Burnout Between Resilience and Engagement in English Learning

The results indicated that students’ demotivation serves as a partial mediator in the relationship between resilience and behavioral engagement, while partially mediated between resilience and agentic engagement in English learning. This suggests that resilience is associated with both behavioral and agentic engagement, and may also be indirectly associated with engagement through its negative association with demotivation.

The relationship among these three variables is in line with the “antecedent-achievement emotions-outcome” framework of the Control-Value Theory (CVT). English learning resilience, a crucial personality trait, can be seen as a distal antecedent for individuals. It can influence achievement emotions by affecting control and value appraisals. Demotivation, as an important dimension of English learning burnout, is a kind of negative achievement emotion. Additionally, English learning engagement is regarded as an important learning outcome in this process. Specifically, in the context of this study, students’ extrinsic value appraisal of English achievement is generally high and remains relatively stable because performance on the Gaokao directly determines opportunities for higher education and social mobility (Fan et al., 2024; Wu et al., 2024). However, students’ control appraisals, namely their judgments of their own academic capabilities, tend to fluctuate in response to daily successes and failures in learning (Pekrun, 2024). Resilience plays a key role in this process. Resilient students maintain positive control appraisals even after setbacks, which may reduce the likelihood of demotivation. When demotivation is alleviated, students’ interest and motivation may be sustained, which may create conditions that support engagement. In contrast, less resilient students are more prone to developing negative control appraisals when encountering difficulties, which may contribute to a state of demotivation. According to CVT (Pekrun, 2006), low control combined with high value increases negative emotions like demotivation, which then undermines engagement. Thus, resilience appears to support engagement not only directly but also by sustaining positive control appraisals and reducing demotivation.

However, the indirect effect via exhaustion was not significant, as exhaustion was not uniquely associated with engagement when accounting for demotivation in the present model.

### 5.3. Limitations

While this mixed-methods analysis reveals meaningful relationships among resilience, burnout, and engagement, these findings are subject to several methodological and contextual limitations. First, while the sample size exceeds 500, meeting the requirements for robust statistical analysis (Hair et al., 2019), the geographical restriction to southeastern China limits the generalizability of the findings. Southeastern China is characterized by abundant educational resources and relatively high levels of educational investment, which may differ considerably from inland or rural regions where such resources are more limited. Thus, further research should expand the research scope to have a more comprehensive understanding of Chinese EFL learners’ resilience, burnout, and engagement in different regions. Second, the cross-sectional design and reliance on a single mediator preclude causal inference. Alternative causal orderings were not tested; the hypothesized direction was derived from CVT and was not compared with competing models, which should be addressed in future research. The single-source self-report data raise concerns about common method variance and social desirability bias; Harman’s single-factor test indicated no dominant factor, but this diagnostic is relatively weak (Podsakoff et al., 2003). Future research could adopt longitudinal designs and multi-informant approaches to strengthen causal and validity claims. Third, students were nested within classes and schools, which may have introduced dependency in the data and underestimated standard errors. Future research could employ multilevel SEM or cluster-robust standard errors to address this issue. Fourth, the measurement model was refined by removing one item in the same sample, which may increase the risk of overfitting; future research should cross-validate the modified model in independent samples. Additionally, the close correlation between exhaustion and demotivation suggests that the non-significant path from exhaustion to engagement should be interpreted with caution. Fifth, the qualitative component was limited in both sample diversity and interpretive depth. Although we have supplemented the sample with additional cases to enhance diversity, the overall sample size remains limited for achieving full saturation. Future research could adopt more purposive sampling strategies to capture a wider range of learner experiences, including cases that deviate from the predominant patterns.

## 6. Conclusions

The current study identified a mediating role of burnout between resilience and engagement in English learning. Specifically, resilience was negatively associated with two dimensions of burnout (i.e., demotivation and exhaustion). Meanwhile, demotivation was negatively associated with engagement, whereas exhaustion did not show a significant unique association with engagement. Additionally, resilience was positively associated with engagement. Moreover, the indirect effect of resilience on engagement via demotivation was significant. These findings contribute to the CVT literature by specifying that demotivation, rather than exhaustion, serves as the primary pathway through which resilience is associated with engagement in the Gaokao EFL context.

## 7. Implications

The current findings offer several practical implications for improving English teaching and learning within the Chinese context. First, despite the encouraging findings that Chinese senior high school students exhibit relatively low burnout alongside high resilience and engagement in English learning, stakeholders must remain vigilant to prevent potential problems. Given the dynamic nature of learner psychology, preventive and interventional measures should be integrated into daily practices. Teachers should proactively diagnose and mitigate environmental stressors such as excessive workload, unrealistic expectations, and insufficient feedback, while also constructing a positive learning ecosystem for EFL learners. Second, given the direct and indirect associations between resilience and engagement identified in this study, both educators and learners should prioritize cultivating resilience. Teachers can guide students to reconceptualize difficulties as growth opportunities through iterative “attempt-adjustment-reattempt” cycles, paired with process-oriented feedback. Finally, since demotivation directly affects engagement and mediates the resilience-engagement relationship, educators should specifically address demotivation, which manifests as erosion of interest and self-efficacy (H. Liu & Zhong, 2022). To cultivate interest, teachers should connect learning to real-life contexts and grant students ownership over their output (e.g., choices between oral presentations or digital posters). To bolster self-efficacy, teachers can deploy AI-assisted tools to break complex learning tasks into achievable, proficiency-matched steps, enabling students to accumulate incremental successes and rebuild confidence.

## Figures and Tables

**Figure 1:**
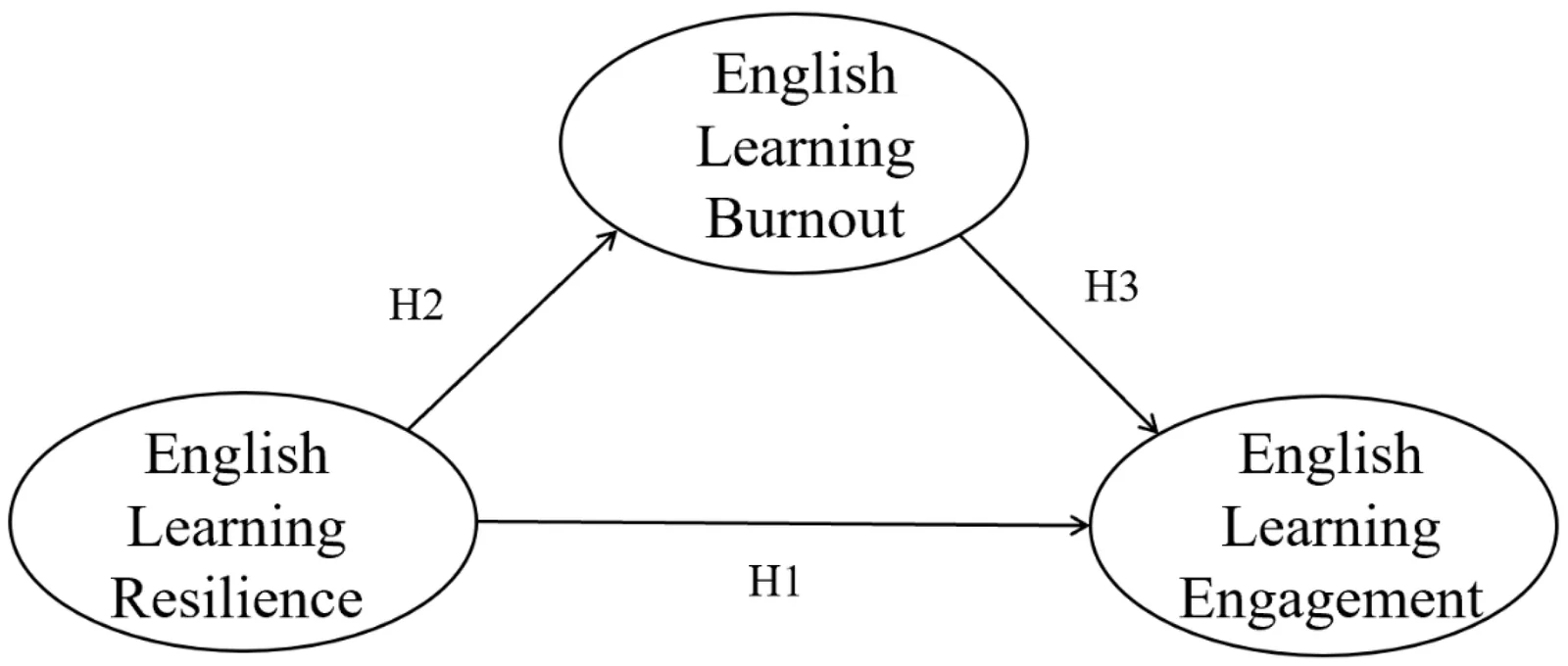
Hypothesized model of the mediation of English learning burnout between English learning resilience and English learning engagement.

**Figure 2:**
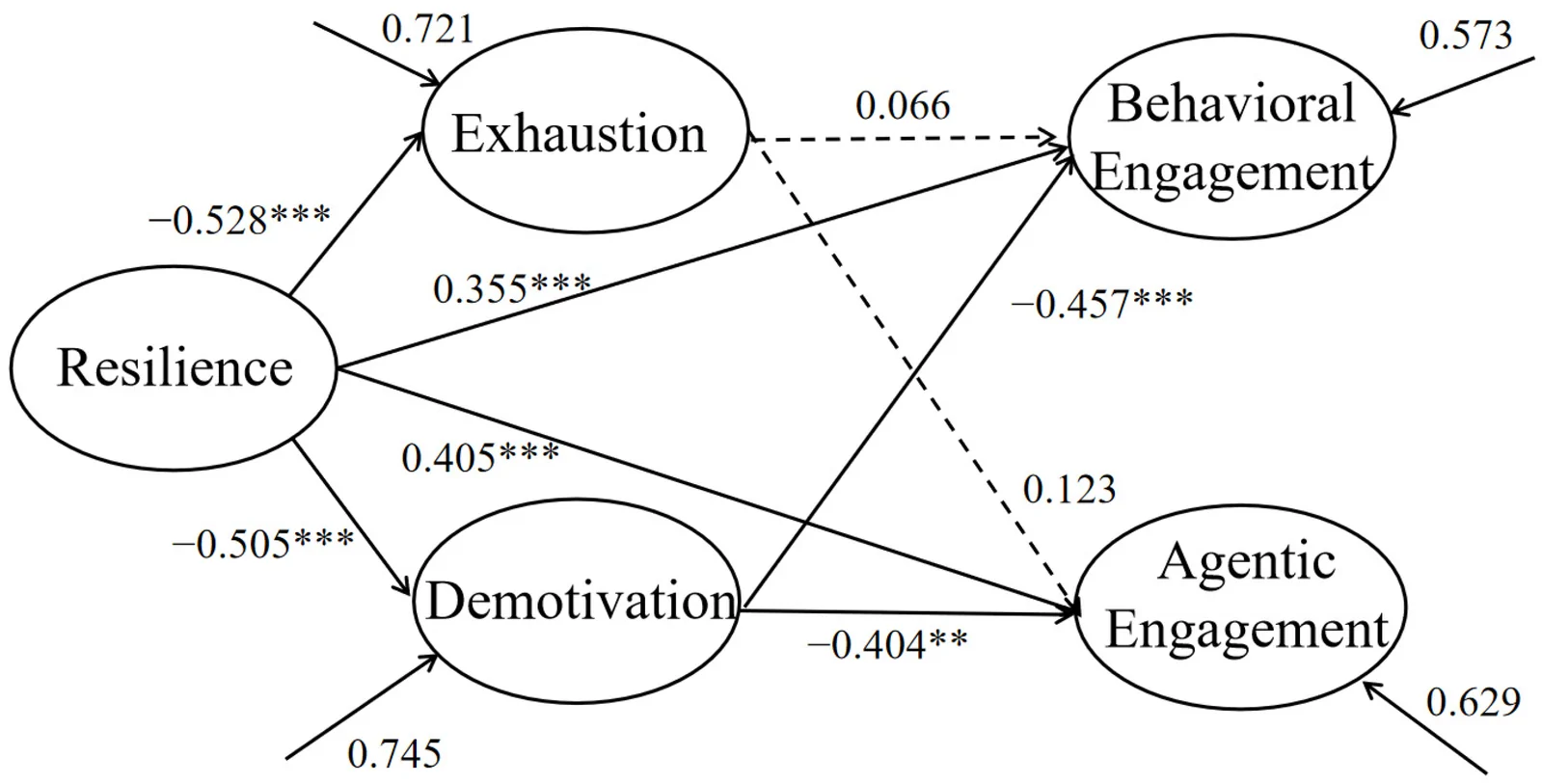
The final SEM. Note. *N* = 637. ** *p* < 0.01; *** *p* < 0.001. Solid lines represent significant relationships and dashed lines represent non-significant relationships.

**Table 1:** The descriptive statistics and correlations among latent variables. (*N* = 637).

Variables	1	2	3	4	5
1. resilience	**0.80**				
2. exhaustion	−0.49 **	**0.85**			
3. demotivation	−0.45 **	0.74 **	**0.74**		
4. behavioral engagement	0.53 **	−0.48 **	−0.51 **	**0.84**	
5. agentic engagement	0.51 **	−0.37 **	−0.40 **	0.60 **	**0.80**
M (SD)	3.65 (0.79)	2.23 (0.92)	2.50 (0.85)	3.81 (0.74)	3.11 (0.94)
Skewness	−0.71	0.66	0.28	−0.76	−0.15
Kurtosis	0.91	0.08	−0.23	1.64	−0.27

Note. *N* = 637. ** *p* < 0.01. The bolded diagonal values represent the square roots of the average variance extracted (AVE).

**Table 2:** Direct and indirect effects of SEM analysis.

Pathway	Β	S.E.	*p*	95% CI	
				Lower	Upper
Resilience → Exhaustion	−0.528	0.044	***	−0.61	−0.436
Resilience → Demotivation	−0.505	0.049	***	−0.595	−0.404
Resilience → BehavioralE	0.355	0.057	***	0.232	0.459
Resilience → AgenticE	0.405	0.059	***	0.286	0.518
Exhaustion → BehavioralE	0.066	0.135	0.624	−0.169	0.361
Exhaustion → AgenticE	0.123	0.12	0.307	−0.082	0.393
Demotivation → BehavioralE	−0.457	0.137	***	−0.773	−0.228
Demotivation → AgenticE	−0.404	0.132	**	−0.696	−0.183
Resilience → Exhaustion → BehavioralE	−0.035	0.071	0.622	−0.191	0.089
Resilience → Exhaustion → AgenticE	−0.065	0.063	0.305	−0.207	0.043
Resilience → Demotivation → BehavioralE	0.231	0.078	**	0.102	0.413
Resilience → Demotivation → AgenticE	0.204	0.075	***	0.087	0.38

Note. *N* = 637; β = Standardized path coefficient, SE = Standard Error, CI = Confidence Interval, BehavioralE = Behavioral engagement, AgenticE = Agentic engagement, ** *p* < 0.01; *** *p* < 0.001.

## Data Availability

The data presented in this study are available on request from the corresponding authors.

## Supplementary Materials

The following supporting information can be downloaded at: https://www.mdpi.com/article/10.3390/bs16101714/s1. Figure S1: Graphical representation of the one-factor model and factor loadings of students’ English learning resilience; Figure S2: Graphical representation of the bi-factor model and factor loadings of students’ English learning burnout; Figure S3: Graphical representation of the bi-factor model and factor loadings of students’ English learning engagement; Table S1: Interviewees’ scores of resilience, burnout, and engagement in English learning; Table S2: Thematic Coding Structure with Supporting Interview Excerpts.

## Appendix A. Questionnaire on High School Students’ Psychological Factors in English Learning

1	I believe I am mentally tough when it comes to English exams.	我相信我能从容应对每次英语考试。
2	I don’t let English study stress get on top of me.	我不会让英语学习压力压倒我。
3	I am good at bouncing back from a poor mark in my English schoolwork.	我善于从英语作业的低分数中恢复过来。
4	I think I am good at dealing with English schoolwork pressures.	我认为我善于处理英语作业所带来的压力。
5	I don’t let a bad mark affect my confidence in English learning.	我不会让糟糕的分数影响我英语学习的信心。
6	I am good at dealing with setbacks in English learning. (e.g., bad mark, negative feedback on my work)	我善于处理英语学习中的挫折（例如，低分、负面反馈）。
7	I feel emotionally drained by my English studies.	英语让我心力交瘁。
8	Studying or attending an English class is really a strain for me.	英语学习或者上英语课对我而言真的是一种折磨。
9	I feel burned out from my English studies.	英语学习让我觉得自己被消耗殆尽。
10	I feel tired when I get up in the morning and I have to face English study another day at school.	早晨起床时，想到新的一天又要面对英语，我会疲惫。
11	I can’t effectively solve the problems that arise in my English studies.	我不能有效地解决英语学习中出现的问题。
12	I have become less interested in my English studies since my enrollment at the high school.	上中学后，我对英语学习的兴趣减少了。
13	I have become less enthusiastic about my English studies.	我对英语学习的热情减少了。
14	I believe that I don’t make an effective contribution to the English classes that I attend.	我不相信我能够在英语课上发挥积极的作用。
15	In my opinion, I am not a student who learns English well.	我觉得我不是个英语学得好的学生。
16	During English class I don’t feel confident that I am effective in getting things done (e.g., taking part in the classroom activities).	英语课上，我对自己正在做的事情（如参加课堂活动）缺少自信。
17	I listen carefully in English class.	我在英语课上认真听讲。
18	I try very hard to learn English in school.	在学校我很努力地学习英语。
19	The first time my English teacher talks about a new topic, I listen very carefully.	每当英语老师讲授新知识时，我都认真听课。
20	I work hard when we start something new in English class.	在英语课上，我会积极参与新的学习内容或活动。
21	I pay attention in English class.	我上英语课时能集中注意力。
22	During the English class, I ask questions.	我会在英语课上提问。
23	I tell the English teacher what I like and what I dislike.	我会告诉英语老师我喜欢学习什么和不喜欢学习什么。
24	I let my English teacher know what I am interested in.	我会告诉英语老师我对英语学习中的哪些方面感兴趣。
25	During English class, I express my preferences and opinions.	我会在英语课上积极表达自己的观点。
26	I offer suggestions about how to make the English class better.	如何让英语课的上课效果更好，我会就此提出我的建议。

## Appendix B. Interview Protocol

1. In your daily English learning, what difficulties have you encountered? Could you give an example and describe the difficulties you have encountered in more detail?
2. When you encounter difficulties in your daily English learning, such as exam pressure, academic performance, or challenging assignments, what do you usually do?
3. Does your interest and enthusiasm for learning English decrease? Under what circumstances does this usually occur?
4. Do you feel tired or exhausted in English learning? What do you think causes this?
5. How actively do you participate in your regular English classes? (e.g., actively answering the teacher’s questions, voluntarily raising your hand to speak, interacting with the teacher, or pointing out mistakes made by the teacher or other students)
6. What kinds of group activities does your English teacher usually organize in class? What role do you typically play in group activities?
7. How do difficulties, fatigue, or reduced interest in English learning affect your classroom engagement? For example, when you feel burned out, you may not engage in class or learning as much as before.
8. How did you successfully resolve these problems? (e.g., through self-regulation or by seeking help from others)
9. After successfully resolving the difficulties encountered in English learning, how did this affect your subsequent classroom engagement?
