# Supplementary material for: Unpacking the Mediating Role of Burnout on the Relationship Between Resilience and Engagement in Chinese Senior High School Students’ English Learning
Source: Behav Sci (Basel). 2026 Sep 22;16(10):1714. doi: 10.3390/bs16101714 (PMC13701281; doi:10.3390/bs16101714)
Supplement: Supplementary file 1 [file behavsci-16-01714-s001.zip › behavsci-4439604-supplementary.pdf]

Supplemental Table S1. Interviewees' scores of resilience, burnout, and engagement in English learning

Student	Gender	Grade	English Learning Resilience	English Learning Burnout	English Learning Engagement
S1*	Male	1	High(M=4.12)	Low(M=1.77)	High(M=4.25)
S2*	Male	1	High(M=3.49)	Low(M=2.11)	High(M=3.33)
S3*	Female	1	Moderate(M=3.01)	Low(M=2.56)	Moderate(M=3.03)
S4	Female	2	High(M=3.80)	Low(M=2.03)	High(M=3.89)
S5*	Female	2	High(M=4.01)	Low(M=1.86)	High(M=4.11)
S6	Male	2	High(M=3.66)	Low(M=2.13)	High(M=3.63)
S7	Male	3	High(M=3.78)	Low(M=1.98)	High(M=3.56)
S8	Female	3	High(M=3.83)	Low(M=2.08)	High(M=4.01)
S9*	Male	3	Low(M=2.16)	Moderate (M=3.08)	High(M=4.01)
S10*	Female	2	Low(M=1.83)	High(M=3.58)	Low(M=2.01)

Note. Interview excerpts from participants marked with an asterisk (*) are included in the main text. Participants were categorized into high, moderate, and low groups based on literature-derived cut-off scores (e.g., Liu & Zhong, 2022; Liu et al., 2023).

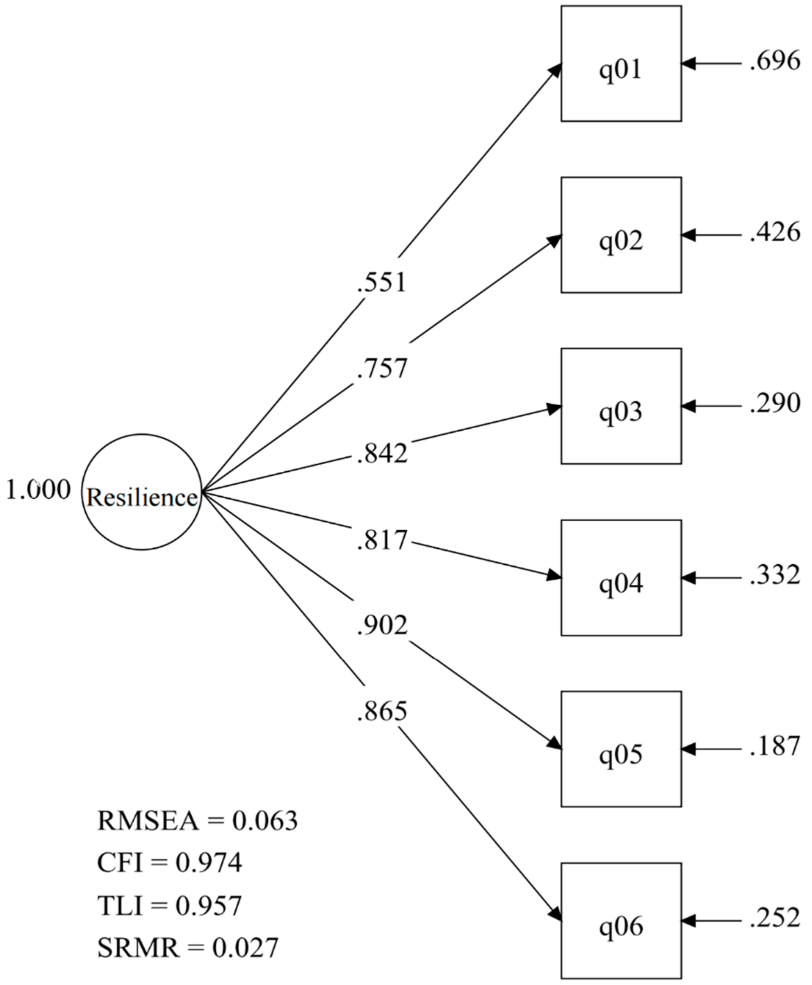

Supplemental Figure S1. Graphical representation of the one-factor model and factor loadings of students' English learning resilience

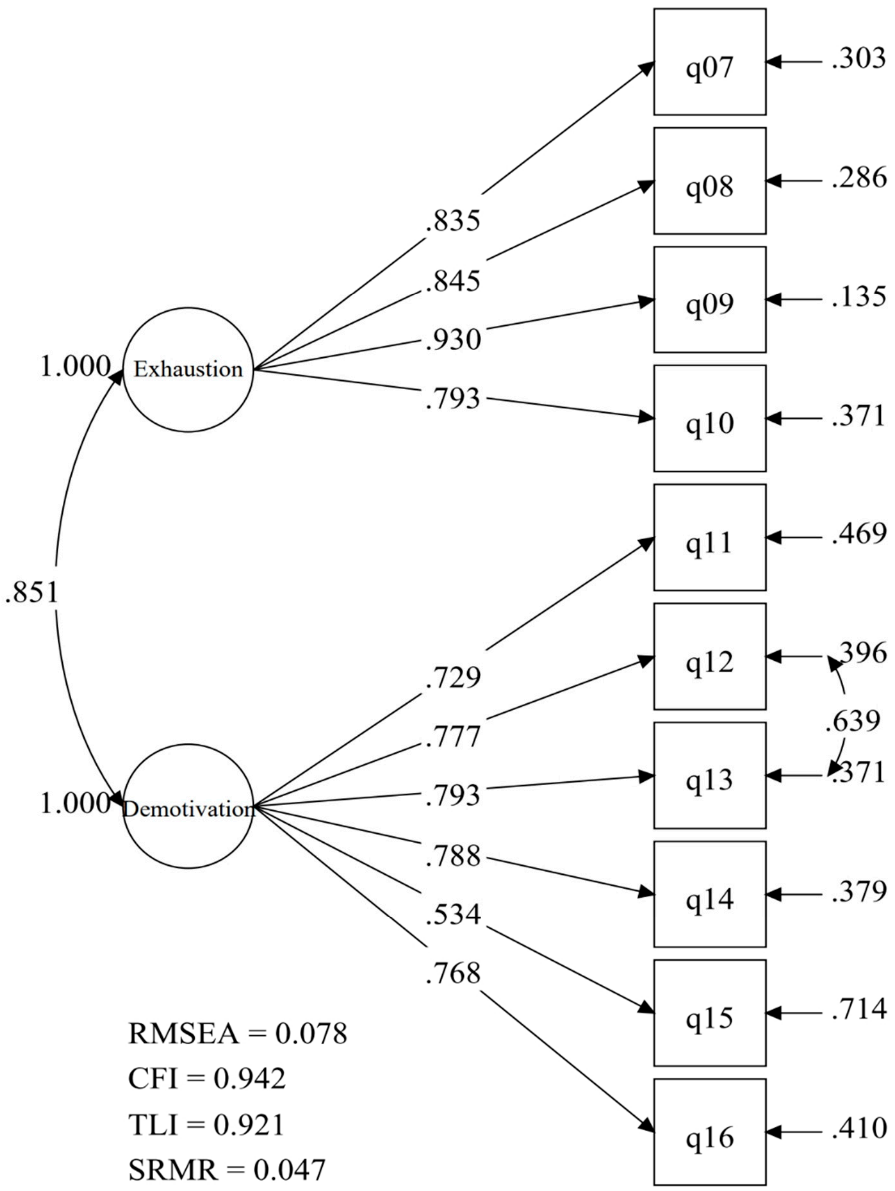

Supplemental Figure S2. Graphical representation of the bi-factor model and factor loadings of students' English learning burnout

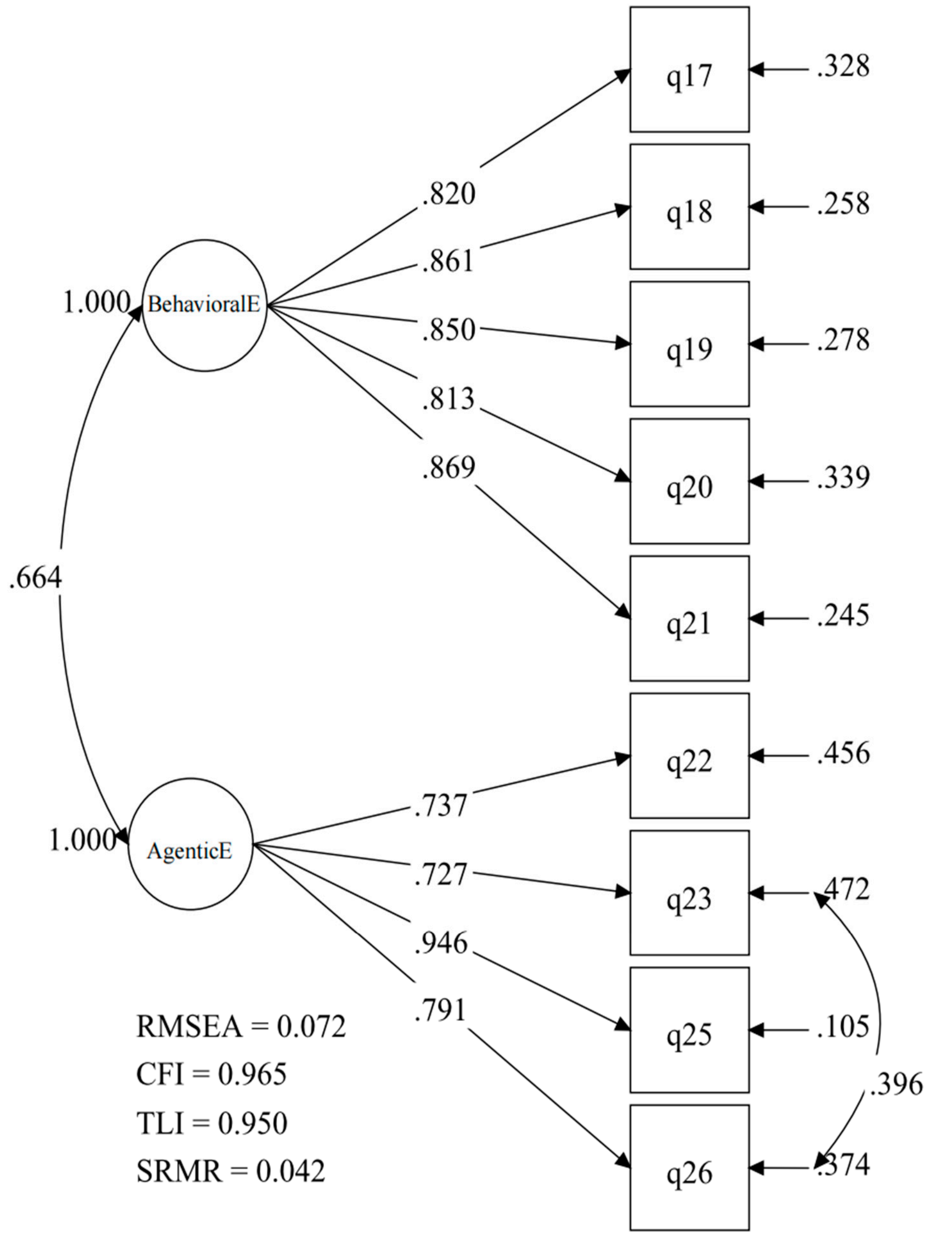

Supplemental Figure S3. Graphical representation of the bi-factor model and factor loadings of students' English learning engagement

Supplemental Table S2. Thematic Coding Structure with Supporting Interview Excerpts

Theme	Initial Code	Interview Sample
Resilience	confidence in own ability to master English	I have reasonable confidence in my abilities and believed that I could master English through my own efforts.
Resilience	successfully tackle problems	After successfully tackling those tricky grammar problems in English learning

Resilience	belief that solutions always outnumber problems	I have always believed that there are always more solutions than problems, I feel confident that I can find a way to solve any challenge I encounter.
Burnout	frustration and resistance toward English learning	I felt frustrated whenever I saw English papers and developed some resistance toward English learning.
Burnout	demotivation and low energy	I sometimes lack energy and motivation in my English learning.
Burnout	demotivation	I began to lose motivation and felt that no matter how hard I tried, it was useless
Engagement	proactive seeking of extra learning materials	I also proactively seek out English papers to work on after school.
Engagement	active classroom participation	taking detailed notes and actively responding to my teacher's questions in class
Engagement	increased time and energy investment after success	regained my motivation to invest more time and energy into learning English.
Direct effect of resilience on burnout	reduced frustration and resistance after resilient actions	My efforts eventually paid off. My English grades gradually improved and I became increasingly motivated.
Direct effect of resilience on engagement	successful overcoming fosters proactive and persistent behaviors	After successfully tackling those tricky grammar problems, I have grown increasingly fond of doing English exercises... I also proactively seek out English papers.
Direct effect of burnout on engagement	loss of enthusiasm leads to avoidance and reduced effort	I gradually lost my enthusiasm later... I did not want to do listening exercises
Mediating role of burnout	more investment after alleviating demotivation	After adjusting my mindset, I regained my drive. I persisted in practicing every day... regained my motivation to invest more time and energy.
